# Concerns Raised by Publication of Antonini et al., “Outcome of Parkinson Disease Patients Affected by Covid‐19”

**DOI:** 10.1002/mds.28180

**Published:** 2020-08-11

**Authors:** Karen G. Raphael

**Affiliations:** ^1^ New York University New York New York USA

As a clinical research scientist and epidemiologist living with Parkinson’s Disease (PD) for more than a decade, it is not unusual to be asked to help translate new research papers and their implications for people living with PD. With stay‐home orders ongoing for nearly 2 months in my NYC home, the coronavirus pandemic is a daily focus. Claims about increased risk of complications for people with PD are speculative, in the absence of data. Thus, I was delighted to finally see some data in the newly accepted report by Antonini et al titled “Outcome of Parkinson Disease Patients Affected by Covid‐10”

Would it confirm the statement in a live webcast in mid‐March by a prominent US PD organization that “PD is a high risk group,” even though most public health reports^1^, ^2^ do not include PD among comorbid conditions associated with serious outcome? It is widely acknowledged that aspiration pneumonia is the leading cause of death in patients with PD. Nevertheless, it is not clear that factors causing people with PD to have elevated risk of aspiration pneumonia similarly cause elevated risk of COVID‐19 pneumonia.

The new report describes the outcome among only 10 people with PD, 2 nursing home residents with advanced disease from the catchment of the Parkinson and Movement Disorders Unit in Padua, Italy, and 8 identified by the Parkinson’s Foundation Centre of Excellence at King’s College Hospital in London, UK. How these patients were identified from the movement disorders centers is unclear. Was systematic screening conducted?

The bottom line from tallying observations on 10 individuals living with advanced PD and suffering from COVID‐19 infection was that 4 of the 10 died. Based on a 40% rate, the authors concluded that “(m)any national and charity guidelines do not list PD or specifically older subjects on advanced therapies as a susceptible group and this information needs to be amended in light of this new data.”

I disagree. Those with mild disease may never present to the medical system, given the absence of known, effective treatments to date. Many individuals in multiple countries test antibody‐positive but were asymptomatic. This is a conundrum for estimating case fatality rates in COVID‐19 infection in general: we have no denominator.

A mortality rate of 40% permits calculation of a 95% confidence limit ranging from 12% to 74% (my calculation). Thus, one can be 95% confident that the interval between 12% and 74% contains the true proportion of advanced PD patients similar to those in their report who, if infected with COVID‐19, would die from their infection. The confidence interval is very large. From this small sample, we cannot conclude that older individuals living with PD who become infected with COVID‐19 have any significantly increased risk of mortality than similar older individuals without PD.

Until additional data are available, it seems unreasonable to assume that most people living with PD at younger ages, with fewer comorbidities and with less advanced disease, are a “high‐risk group” for adverse outcomes in the event of COVID‐19 infection. In the absence of persuasive data, care should be taken to avoid adding unnecessary stress to people living with PD by making such an assertion. As noted recently,^3^ public health risk mitigation strategies present unavoidable stressors for many people with PD living through the pandemic. Psychological stress is associated with symptom exacerbation. Please do not add any unnecessary stress that would occur if we were to assume that case fatality rates drawn from a tiny and unrepresentative sample of elderly adults living with advanced PD inform us about the still‐unknown risk to the larger community of people living with PD.

## Documentation of role

Manuscript preparation, writing of draft

## Financial Disclosures past 12 months

KG Raphael has received honoraria from the World Parkinson Congress and grant support from the US National Institutes of Health (R01 DE04522). She is employed full‐time as a tenured Professor at New York University.

## References

[1] Abduljalil JM , Abduljalil BM . Epidemiology, genome, and clinical features of the pandemic SARS‐CoV‐2: a recent view. New Microbes New Infect 2020;35:100672. 32322400 10.1016/j.nmni.2020.100672 PMC7171182

[2] Richardson S , Hirsch JS , Narasimhan M , et al. Presenting characteristics, comorbidities, and outcomes among 5700 patients hospitalized with COVID‐19 in the New York city area. JAMA 2020;323(20):2052–2059. 10.1001/jama.2020.6775 PMC7177629 32320003

[3] Helmich RC , Bloem BR . The Impact of the COVID‐19 pandemic on Parkinson’s disease: hidden sorrows and emerging opportunities. J Parkinsons Dis 2020;10(2):351–354. 32250324 10.3233/JPD-202038 PMC7242824

